# Determinants And Prognostic Value of Sepsis-Associated Encephalopathy: Insights From The Prospective Multicentre Outcomerea Registry

**DOI:** 10.1186/2197-425X-3-S1-A48

**Published:** 2015-10-01

**Authors:** J Poujade, R Sonneville, M Garrouste-Orgeas, B Souweine, E Azoulay, M Darmon, E Mariotte, L Argaud, F Barbier, D Goldgran-Toledano, G Marcotte, D Anne-Sophie, S Jamali, V Laurent, S Ruckly, JF Timsit, OUTCOMEREA

**Affiliations:** Bichat-Claude Bernard University Hospital, Intensive Care Medicine, Paris, France; Saint-Joseph Hospital, Intensive Care Medicine, Paris, France; Gabriel Montpied University Hospital, Intensive Care Medicine, Clermont-Ferrand, France; Saint-Louis University Hospital, Intensive Care Medicine, Paris, France; Saint-Etienne University Hospital, Intensive Care Medicine, Saint-Priest-en-Jarez, France; Lyon University Hospitals, Intensive Care Medicine, Lyon, France; Orleans Hospital, Intensive Care Medicine, Orleans, France; Gonesse Hospital, Intensive Care Medicine, Gonesse, France; Béclère University Hospital, Intensive Care Medicine, Clamart, France; Dourdan Hospital, Dourdan, France; Versailles Hospital, Intensive Care Medicine, Le Chesnay, France

## Introduction

Severe sepsis and septic shock are frequently complicated by an encephalopathy ranging from confusion to coma (sepsis-associated encephalopathy, SAE).

## Objectives

To characterize risk factors and the prognostic contribution of SAE in adult patients.

## Methods

We conducted a retrospective analysis of all patients with severe sepsis or septic shock included in the French prospective multicenter (n = 12 ICUs) OUTCOMEREA database between 1997 and 2014. We excluded patients with acute brain injury as a cause of ICU admission. SAE was defined by a Glasgow coma scale < 15 at ICU admission. Independent factors associated with SAE were identified using multivariate logistic regression analysis. Data are presented in median (IQR) or number (%).

## Results

Among the 18713 patients of the cohort, 3486 patients with severe sepsis or septic shock at admission were identified. Among them, 137 patients with primary acute brain injury were excluded. Data from 3349 patients (age 66 (54-77) years, male sex 61%, medical admission 71%) were analyzed. Overall, 1586 (47%) patients had evidence of SAE on ICU admission. SAE was more frequently observed in patients with bloodstream infections (88% vs. 79%, p < 0.01) and in patients with pneumonia (45% vs 33% p < 0,01). Compared to patients without SAE, patients with SAE had higher SOFA scores (10 (7-12) versus 7 (4-9) p < 0.01), a higher length of stay in the ICU (8 (3-17) days vs. 6 (3-14) days p < 0.01), a higher ICU mortality (40% vs. 18%, p < 0.01) and a higher hospital mortality (52% vs. 29%, p < 0.01).

In multivariable logistic regression analysis, after adjustment for type of admission, immune status, history of psychiatric disorder, chronic alcohol consumption, and SAPS II score, the following parameters, identified on the day of ICU admission, remained independently associated with SAE

## Conclusions

SAE is observed in about half of adult patients with sepsis at admission to the ICU and is associated with poor outcome. Our analysis identified potentially modifiable factors associated with SAE, including severe metabolic disturbances and pharmacologic agents commonly used at the early phase of sepsis management. Those factors may represent interesting therapeutic targets to lower the prevalence, the intensity and/or the duration of the SAE in critically ill patients.

**Table 1:** Factors associated with SAE, multivariate analysis.

Variable	OR	95%CI inf	95% CI sup	p
Hypercapnia	1.375	1.139	1.660	0.0009
Fluoroquinolone	3.328	1.386	7.989	0.0071
Benzodiazepines	1.546	1.168	2.046	0.0023
Steroids	1.378	1.148	1.654	0.0006
Hypernatremia	2.220	1.540	3.201	<.0001
Sedative drugs	1.947	1.510	2.510	<.0001

